# The Relationship between Glucosinolates and the Sensory Characteristics of Steamed-Pureed Turnip (*Brassica Rapa* subsp. *Rapa* L.)

**DOI:** 10.3390/foods9111719

**Published:** 2020-11-23

**Authors:** Nurfarhana Diana Mohd Nor, Stella Lignou, Luke Bell, Carmel Houston-Price, Kate Harvey, Lisa Methven

**Affiliations:** 1 Department of Early Childhood Education, Faculty of Human Development, Sultan Idris Education University, Tanjong Malim 35900, Perak, Malaysia; farhanadiana@fpm.upsi.edu.my; 2 Sensory Science Centre, Department of Food and Nutritional Sciences, University of Reading, Whiteknights, Reading RG6 6DZ, UK; s.lignou@reading.ac.uk; 3 School of Agriculture, Policy & Development, University of Reading, Whiteknights, Reading RG6 6EU, UK; luke.bell@reading.ac.uk; 4 School of Psychology and Clinical Language Sciences, University of Reading, Early Gate, Whiteknights, Reading RG6 6AL, UK; c.houston-price@reading.ac.uk (C.H.-P.); k.n.harvey@reading.ac.uk (K.H.)

**Keywords:** glucosinolates, turnip, *Brassica*, bitter taste, *Brassicaceae*, vegetable

## Abstract

Glucosinolates (GSLs) are phytochemical compounds that can be found in *Brassica* vegetables. Seven separate batches of steamed-pureed turnip were assessed for GSL content using liquid chromatography mass spectrometry (LC-MS) and for sensory attributes by sensory profiling (carried out by a trained sensory panel). Twelve individual GSLs, which included 7 aliphatic, 4 indole and 1 arylaliphatic GSL, were identified across all batches. There were significant differences in individual GSL content between batches, with gluconasturtiin as the most abundant GSL. The total GSL content ranged from 16.07 to 44.74 μmol g^−1^ dry weight (DW). Sensory profiling concluded there were positive correlations between GSLs and bitter taste and negative correlations between GSLs (except glucobrassicanapin) and sweet taste. The batches, which had been purchased across different seasons, all led to cooked turnip that contained substantial levels of GSLs which were subsequently all rated as bitter.

## 1. Introduction

*Brassica* vegetables such as turnip, cabbage, broccoli and cauliflower are rich with sulphur-containing glucosinolate compounds (GSLs) [1]. These compounds are water-soluble and have a role in plant defence against pests and diseases [2]. GSLs can be structurally classified into aliphatic, arylaliphatic and indole types [1]. Kim and Park [3] discussed that the degradation products of GSLs possess anticarcinogenic properties, reducing risks of certain cancers in humans. Glucoraphanin, glucobrassicin and gluconasturtiin are among the GSLs that have hydrolysis products shown to have anti-cancer properties, and these are all found in turnip [4].

GSLs are, amongst other compounds, partly responsible for the taste characteristics of *Brassica* vegetables. Individual GSLs such as sinigrin, gluconapin, progoitrin and neoglucobrassicin have been associated with bitter taste [5,6]. Furthermore, Bell et al. [7] reported that GSLs were also correlated with earthy, pepper, mustard flavour and pungency in rocket varieties (*Eruca sativa* Mill.).

GSL contents in *Brassica* vegetables are influenced by many factors, such as environmental conditions and genetic variability between cultivars. The abundance of GSLs in plants is varied, depending on the type of plant species, developmental stage and plant part (root, shoot, seeds and leaves) [8,9,10]. Concerning cultivars, Kabouw et al. [10] showed that there was a significant difference in GSL content between white cabbage cultivars (*Brassica oleracea* var. *capitata* L.), and Zhu et al. [11] reported significant differences in GSL content between pak choi cultivars. In addition, nutrient supply contributes to the concentration of GSLs in plants. GSL content increases with an adequate supply of sulphur [12], however nitrogen in the absence of sulphur and also selenium supply have been shown to result in a decrease of GSL content [13,14], whereas nitrogen with a sufficient sulphur supply may either increase GSL content or have no effect [14]. Such variations in GSLs can lead to distinctive sensory characteristics of *Brassica* vegetables [15], which are thought to influence their consumption frequency [16].

GSL content in *Brassica* vegetables is also affected when they are handled and prepared before consumption. GSLs undergo hydrolysis to produce breakdown products when the plant cells are wounded [17]. Preparation processes, including cooking and cutting, trigger myrosinase enzymes in plant cells to hydrolyse GSLs and produce isothiocyanates (ITCs) plus other breakdown products; including nitriles, thiocyanates, epithionitriles, oxazolidine-2-thiones and epithioalkanes [15]. A review by Nugrahedi [18] concluded that boiling and blanching significantly reduced GSL content in *Brassica* vegetables due to leaching of compounds. On the other hand, steaming, microwaving and stir-frying may limit GSL loss compared to boiling.

Turnips (*Brassica rapa* subsp. *Rapa* L.) are a traditional vegetable grown in the UK that are no longer frequently consumed by UK consumers in comparison to other *Brassica* vegetables, such as broccoli, cauliflower and cabbage. In 1992, turnip (together with swede) accounted for 5400 hectares of production whereas this had dropped to less than 2700 hectares by 2017. Although the field area for cauliflower fell over the same period, it remained higher than turnip at over 9200 hectares in 2017 [19]. However, turnip could provide a beneficial source of glucosinolates if incorporated more regularly into the diet. As a vegetable that is predominantly consumed cooked, it is the GSL and sensory profile of cooked turnips that are of relevance to the consumer.

Realising that GSL content in commercial turnip may vary between cultivars, growth conditions, seasons and cooking batches, the aim of this study was to evaluate the variability in GSL content and resulting differences in sensory perception, as purchased commercially and as the vegetable would be consumed by consumers. Numerous research papers concerning *Brassica* vegetables focus on the raw vegetable rather than the material as consumed, and where studies focus on cooking, they recommend minimal cooking to preserve GSL content. Minimal processing is not suitable for a hard root vegetable such turnip, and therefore, it is important to establish whether more rigorous cooking and preparation does successfully deliver beneficial GSL to consumers. To achieve our aim, seven batches of steamed-pureed turnip were prepared and subsequently analysed for GSLs (identification and quantification using liquid chromatography mass spectrometry; LC-MS) and sensory profile (trained sensory panel). The hypothesis was that each batch of steamed-pureed turnip would contain substantial amounts of GSLs and have a perceivable bitter taste, regardless of any differences between batches.

## 2. Materials and Methods 

### 2.1. Turnip Sample and Preparation

Seven batches of steamed-pureed turnip were used in this study. Steaming was chosen rather than boiling to reduce loss of GSLs leaching into cooking water. Pureeing was chosen to produce a homogenous sample, and also steam and puree are among many methods used to prepare turnips at home. Turnips (grown in the UK and the Netherlands) were bought from two local stores in Reading (UK), from December 2015 to June 2016, and each batch was cooked on a different day (Table 1).

The root was used in the preparation of the samples; prior to cooking, turnips were peeled, and stems and tails removed, then washed and sliced to a thickness of approximately 0.5 cm. Between 8.2 and 13.6 kg raw turnips were used to make each batch of cooked turnip. For each cooking cycle, approximately 2.4 kg of sliced turnips were placed into an electric 3-tier steamer (Tefal; 800 g in each tier), with 1 L of water added to the base of the steamer and steamed for 25 min. Sliced turnips from tier 1 were transferred to tier 3 and vice versa (to ensure equal heat circulation), water was added again up to 1 L and steamed for another 25 min to ensure the root was soft enough to be blended. The internal temperature of the steamer was ~64 °C. Turnips were then blended using a hand blender (Russell Hobbs) for approximately 5 min until the texture was smooth. All cooked turnips were then placed into plastic containers, labelled, and stored in a freezer at −18 °C.

Prior to GSL extraction, samples were frozen (−80 °C) and then freeze-dried for 5 days (Stokes freeze dryer, F.J Stokes Corporation, Philadelphia, USA). The dried samples were ground (pestle and mortar) and then sieved (20 mesh) to ensure a fine powder.

### 2.2. Reagents and Chemicals

All chemicals used were of LC-MS grade and purchased from Sigma-Aldrich (Poole, UK), unless otherwise stated.

### 2.3. Glucosinolates Extraction

The extraction method was adapted from [20]. Three replicates of each batch were prepared as follows: 40 mg of ground steamed-pureed turnip powder was heated in a dry-block at 75 °C for 2 min to ensure inactivation of any remaining active myrosinase enzyme. Preheated 70% (*v*/*v*) methanol (1.2 mL; 70 °C) was added and the sample placed in a water bath for 20 min at 70 °C. Samples were then centrifuged for 10 min (10,000 rpm, 18 °C) to collect loose material into a pellet. The supernatant was then filtered through 0.22 μm Acrodisc syringe filters with Supor membrane (hydrophilic polyethersulfone; VWR, Lutterworth, UK) and frozen (−80 °C) in Eppendorf tubes until analysis by LC-MS.

### 2.4. LC-MS Analysis

LC-MS analysis method was adapted from [21]. Sinigrin hydrate was used as an external reference standard for quantification of GSL compounds. Preparation was as presented by Jin et al. [22]. LC-MS analysis was performed in the negative ion mode on an Agilent 1260 Infinity Series LC system (Stockport, UK) equipped with a binary pump, degasser, autosampler, column heater, diode array detector, coupled to an Agilent 6120 Series single quadrupole mass spectrometer. Separation of compounds was achieved on a Gemini 3 μm C_18_ 110 Å (150 × 4.6 mm) column (with Security Guard column, C_18_; (4 mm × 3 mm); Phenomenex, Macclesfield, UK). GSLs were separated during a 40 min chromatographic run, with 5 min post-run sequence. Mobile phases consisted of ammonium formate (0.1%; A) and acetonitrile (B) with the following gradient timetable: (i) 0 min (A-B, 95:5, *v*/*v*); (ii) 0–13 min (A-B, 95:5, *v*/*v*); (iii) 13–18 min (A-B, 40:60, *v*/*v*); (iv) 18–26 min (A-B, 40:60, *v*/*v*), 26–30 min (A-B, 95:5, *v*/*v*); (v) 30–40 min (A-B, 95:5, *v*/*v*). The diode array detector recorded spectra at 229 nm. The flow rate was optimised for the system at 0.4 mLmin^−1^, with a column temperature of 30 °C, with 25 μL of sample injected into the system. Quantification was conducted at a wavelength of 229 nm.

MS analysis settings were as follows: API-ES was carried out at atmospheric pressure in negative ion mode (scan range m/z 100–1500 Da). Nebulizer pressure was set at 50 psi, gas-drying temperature at 350 °C, and capillary voltage at 2000 V.

Compounds were identified using MS through both spectra available in the literature [23,24] or from GSL standards in our own laboratory and by comparing relative retention times with those published in the literature [25]. Semi-quantification was carried out using UV absorbance (diode array detector; DAD) peak area data and relating that to the external sinigrin standard (regression: y = 26.7X + 52.6; *r*^2^ = 0.998). Relative response factors (RRFs) were used in the calculation of GSL concentrations where available [23]; however, they were assumed to be 1.00 if such data was not available in the literature [25] or from our laboratory standards. All data were analysed using Agilent OpenLAB CDS ChemStation Edition for LC-MS (Agilent, version A.02.10).

### 2.5. Sensory Analysis

Sensory analysis was carried out by nine sensory trained panellists, each with a minimum of six months experience, using sensory profiling. The panel developed a consensus vocabulary for the seven batches of steamed-pureed turnip concerning aroma, taste and flavour (Table 2). Spinach, mashed potato, sucrose (granulated sugar) and quinine sulphate solutions were used as references to help the panel to standardise the vocabulary. During duplicate sample evaluations, samples were presented in a balanced sequential order, and each characteristic was scored on a line scales (0–100), using Compusense Cloud Software (Ontario, Canada). Line scales were unstructured except for the sweet and bitter attributes where a structured scale was used. Table 2 shows the levels of reference standards used for these two attributes. The panel tasted and scored the reference standards; their mean values for these standards were used as anchors on the scale. For sweet, the anchor positions for the four standards were 13.8, 29.1, 57.6 and 80.6, respectively. For bitter taste, the anchor positions were 8.1, 23.0, 38.9, 63.2 and 82.6, respectively. Evaluation sessions were conducted in a sensory room within the Sensory Science Centre at the Department of Food and Nutritional Sciences, Reading, UK. Each panellist sat in an individual booth equipped with artificial daylight and with room temperature controlled (approximately 22 °C).

### 2.6. Statistical Analysis

The analytical results presented are the mean of three replicates (*n* = 3) for each sample. One-way ANOVA was used for comparison of GSL content between batches of steamed-pureed turnip. A principal component analysis (PCA) was carried out to relate GSLs with sensory characteristics. GSL data were projected onto the PCA with the mean sensory data as supplementary variables; Pearson’s correlation was used. These tests were performed by using XLStat (Addinsoft, Paris, France).

Sensory profile data were tested using two-way ANOVA in SENPAQ (Qi Statistics Ltd., Reading, UK) where the main effects (sample and assessor) were tested against the sample by assessor interaction, with sample as fixed effect and assessor as random effect. All significant differences between samples were assessed by using Tukey’s HSD post hoc test at a significance level of 5%.

## 3. Results

### 3.1. Identification and Quantification of Glucosinolates

**Figure 1:**
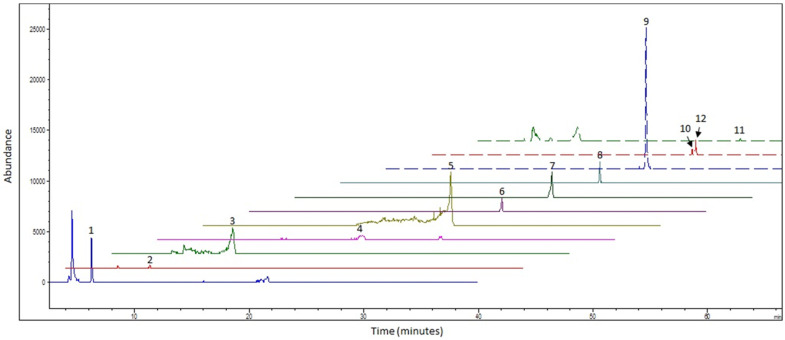
Example LC-MS extracted ion chromatogram of glucosinolates from steamed-pureed turnips. Peak identity: (1) progoitrin; (2) glucoalyssin; (3) gluconapin; (4) 4-hydroxy-glucobrassicin; (5) glucobrassicanapin; (6) glucoerucin; (7) glucobrassicin; (8) glucoberteroin; (9) gluconasturtiin; (10) 4-methoxyglucobrassicin; (11) Gluconapoleiferin and (12) Neoglucobrassicin, corresponding with glucosinolates listed in Table 3 and their extracted mass ion.

Twelve individual GSLs were detected across all batches of steamed-pureed turnip (Figure 1), and the concentration of each of GSL varied significantly between batches (Table 3). There were 7 aliphatic GSLs (progoitrin, glucoalyssin, gluconapin, glucobrassicanapin, gluconapoleiferin, glucoerucin, and glucoberteroin), 4 indole GSLs (4-hydroxyglucobrassicin, glucobrassicin, 4-methoxyglucobrassicin, and neoglucobrassicin) and 1 arylaliphatic GSL (gluconasturtiin). Glucoalyssin was only detected in batches B1 and B2, while no glucoerucin was detected in B5. Gluconasturtiin was the most abundant GSL across all batches. Total GSL concentration ranged from 16.07 to 44.74 μmol g^−1^ DW.

### 3.2. Sensory Characteristics

Table 4 summarises the mean sensory characteristic scores for the seven batches of steamed-pureed turnip. There was a significant difference in wet aroma where batch B2 had a higher score than B7. No other aroma characteristics were significantly different between batches.

For taste characteristics, there was a significant difference in bitter taste between batches, where batch B2 had the highest intensity for bitter taste, whereas B1 and B4 were significantly less intense. All batches were perceived as bitter with mean ratings varying between 30 and 53 in bitter taste intensity of the 0.0002% and below the 0.0004% quinine standard used. Sweetness did not vary significantly between batches; the range of mean scores were between 26 and 35 on the 100-point scale, being in the region of sweetness of the 1% sucrose standard used.

Significant differences between batches can be found for tannin and apple flavour. B2 was significantly higher than B1, B3, B4 and B5 for tannin flavour. B5 was significantly higher than B2, B6 and B7 in terms of apple flavour. There were no significant differences between batches for other characteristics.

### 3.3. Principal Component Analysis (PCA)

**Figure 2:**
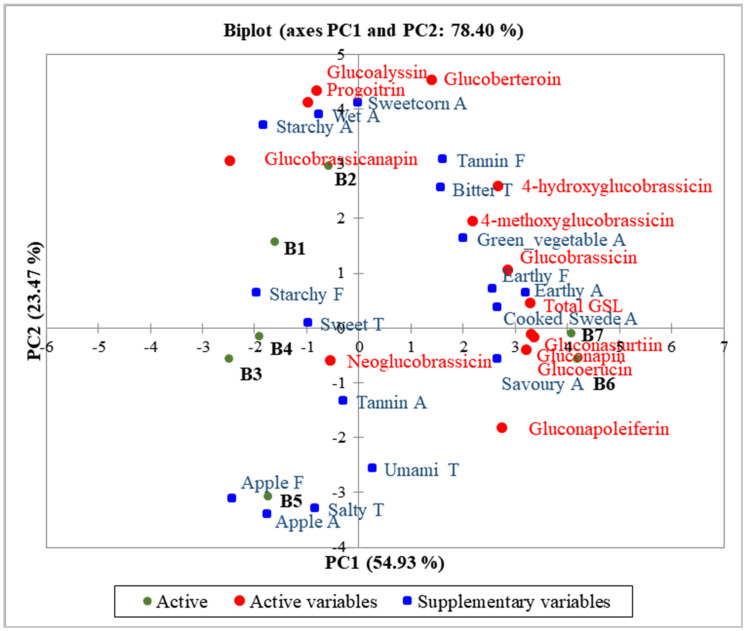
PCA biplot of glucosinolate compounds in 7 batches of steamed-pureed turnip (B1 to B7), with mean ratings of sensory attributes fitted onto the plot as supplementary variables. Abbreviations: A, aroma; T, taste; F, flavour.

Principal component analysis (PCA) of the GSL data was carried out to demonstrate the batch separation according to GSLs, and onto this map the sensory data was fitted as supplementary data in order to investigate any correlation of the GSLs with the sensory characteristics (Figure 2). Dimensions 1 and 2 recovered over 78% of the variance in the data. Total GSL and many of the individual GSLs were predominantly located on the right side of PC1, located alongside turnip batches B6 and B7. PC2 was highly correlated with gluoberteroin (*r* = 0.88) and glucoalyssin (*r* = 0.84).

The position for the total GSL content strongly correlated with PC1 (*r* = 0.98) and also to many of the individual GSLs: gluconapin (*r* = 0.99, *p* < 0.001), gluconasturtiin (*r* = 0.98, *p* < 0.001), glucoerucin (*r* = 0.97, *p* < 0.001), 4-hydroxyglucobrassicin (*r* = 0.82, *p* = 0.03), glucobrassicin (*r* = 0.78, *p* = 0.04) and gluconapoleiferin (*r* = 0.77, *p* = 0.04). However, 4 other GSLs strongly correlated with each other and with dimension PC2: glucoberteroin (*r* = 0.88), glucoalyssin (*r* = 0.84), progoitrin (*r* = 0.80) and glucobrassicanapin (*r* = 0.59).

There was a clear separation of groups of sensory characteristics on the PC biplot. Earthy (aroma and flavour), cooked swede aroma and savoury aroma were positioned to the right of PC1 and negatively correlated with sweet taste. Bitter taste and tannin flavour were positioned in the top right quadrant of the plot and negatively correlated with apple (aroma and flavour).

As expected, many of the GSLs correlated with bitter taste. The total GSL content was positively, but not significantly, correlated with bitter taste (*r* = 0.47, *p* = 0.29). Of the 12 GSLs quantified, one, glucobrassicanapin, had clearly no association with bitter taste (*r* = 0.033, *p* = 0.94) whereas the correlation coefficient between the other GSLs and bitter taste varied between 0.30 and 0.75. The only significant correlation was 4-methoxyglucobrassicin (*r* = 0.82, *p* = 0.02), while glucobrassicin also had a strong positive correlation (*r* = 0.75, *p* = 0.052), despite the levels of these two GSLs not being particularly high in the turnip batches, indeed very low for 4-methoxyglucobrassicin (Table 3). Such correlations cannot prove which of these GSLs have the greatest contribution to bitter taste, but they do support the hypothesis that the GSLs in turnip contribute to bitter taste. Bitter taste will suppress sweet taste, so it was as expected that all GSLs (except glucobrassicanapin) were negatively correlated with sweet taste (*r* = −0.55 to *r* = −0.01).

B1 and B2 were negatively correlated with B6 and B7; B1 and B2 were separated from B3, B4 and B5 along PC2. Moreover, B6 and B7 were separated from the other batches along PC1, and this was driven by the higher level of total GSL and particularly 4-hydroxyglucobrassicin, 4-methoxyglucobrassicin, glucobrassicin, gluconasturtiin, gluconapin and glucoerucin. These 2 batches were indeed the most bitter tasting, along with B2, which although not as high in total GSL, was highest in glucobrassicanapin. PC2 particularly separated B5 from B2, where B5 was particularly low in all GSLs and higher in apple (aroma and flavour).

## 4. Discussion

Twelve individual GSLs were detected across all batches. The total GSL content ranged from 16.07 to 44.74 μmol g^−1^ DW with mean value of 27.37 μmol g^−1^ DW. The total content is comparable to findings reported by Zhang et al. [26], (16.4 to 31.4 μmol g^−1^ DW), but lower than those reported by Lee et al. [4], (117.05 μmol g^−1^ DW). Zhang et al. [26] freeze dried the raw turnip roots rather than cooked turnip as in the present study; however, both studies later incubated at 75 °C before extraction with methanol. The results remain comparable as the steaming of turnip in the current study would denature the myrosinase enzyme and limit transformation to hydrolysis products. Other reasons might be because of the similarity in environmental factors that both studies have, as the turnips were sown across different seasons, which then would yield similar GSL content. Contradictory to the Lee et al. [4] study, the turnips were sown and grown in a controlled environment (i.e., temperature-controlled) to minimise seasonal differences, hence the large difference in the GSL content.

In the present study, aliphatic GSLs were the most abundant, representing 48.6% of total GSL content, followed by 45.6% of arylaliphatic GSL and 5.8% of indole GSL. These results are in agreement with other studies which confirm that these compounds are common GSLs in turnip varieties [4,26,27,28]. Gluconasturtiin was the dominant GSL (45.6%), ranging from 8.96 to 19.81 μmol g^−1^ DW, with a mean value of 12.48 μmol g^−1^ DW. This GSL compound has previously been shown to be the most abundant in turnip greens [28] and turnip roots [26].

There were significant differences in each individual GSL between batches and this is expected as the turnips were bought on different days, across different seasons, and from a variety of suppliers. Although they were all “purple top” turnips, they were potentially of different cultivars. Type of cultivar will affect GSL content; indeed, Kim et al. [29] reported that the GSL content of turnip seeds varied significantly between 12 cultivars. There are many other factors that could also contribute to variability. Kim et al. [30] reported that GSL content in turnip is dependent on harvest times. Subsequent research papers have noted that, in addition to harvest time, growth season could also result in the GSL variation [26,31]. Environmental conditions of different growing sites, such as soil pH, can influence GSL content too [32]. Our PCA plot showed that batches B1 and B2 were similar, as were B4 and B5, and B6 and B7. These similarities can be explained by the month the turnips were purchased. Turnips for batches B1 and B2 were purchased in autumn/winter season, and they were negatively correlated in terms of GSL content and sensory characteristics, with B6 and B7, which were bought in spring/summer season. Although turnips for batch B3 were purchased in a different season from B4 and B5, these three batches were correlated with each other, in terms of GSL content and sensory characteristics. It could be speculated that these three batches may be from the same cultivar of turnip, and the cultivar effect is greater than season effect; however, this cannot be concluded as the turnip cultivar was not controlled for in this study.

In summary, the significant differences in GSL content between cooked turnip batches in this study might be caused by differences in cultivars, seasons or growth conditions. Turnips sold in the UK come from many different countries with different growth conditions. Therefore, variation in GSL content at the point of consumption is expected from turnips purchased in the UK supermarkets at different times of year.

GSLs are among the compounds that are responsible for the sensory characteristics of *Brassica* vegetables. As noted in (Figure 2), most of the GSLs were positively (although not significantly) correlated with bitter taste, the strongest correlations being with 4-methoxyglucobrassicin and glucobrassicin. Although Helland et al. [33] also found 4-methoxyglucobrassicin to be related to the bitterness of swede and turnip, the levels of this compound in the current study were very low. Glucobrassicin was present at higher levels (0.65 to 1.19 μmolg^−1^ dry weight) and was clearly correlated with most bitter turnip batches (B2, B6 and B7). Glucobrassicin has previously been reported to cause bitter taste, alongside 4-hydroxyglucobrassicin, progoitrin, gluconapin and neoglucobrassicin, in turnip, swede, rocket, broccoli and cauliflower, [33,34,35] which is consistent with the current study where all were positively correlated with the bitter taste in turnip (*r* = 0.33 to *r* = 0.75). The GSL in highest abundance in the cooked turnip was gluconasturtiin; this has a positive but relatively weak correlation to bitter taste (*r* = 0.43). Although this finding does not confirm a relationship between gluconasturtiin and bitterness, it was present at high levels in all batches (compared to other GSLs), and all batches were perceived to be bitter. Bladh et al. [36] previously concluded that it was a hydrolysis product of gluconasturtiin, phenethyl isothiocyanate, that had a strong bitter taste. Although GSLs are accepted to impart bitterness, the correlation between GSLs and perception of bitterness does not confirm causality. Bell et al. [37] reviewed the relationship between GSLs and bitterness and noted that there very few studies where GSLs have been isolated and rated by sensory panels; one specific exception being sinigrin where bitter taste thresholds have been reported. Relating bitter taste to specific GSLs in *Brassica* samples is limited by the high correlation between the quantities of individual GSLs. This Bell et al. [37] review also noted that inconsistencies in relating GSLs to bitter taste can also arise from differences in preparation and cooking methods between studies. In addition, hydrolysis product of GSLs are often not quantified, and therefore, their contribution to bitterness is often not accounted for. A further review by Wieczorek et al. [38] concluded that inconsistencies between studies can also result from differences in consumers’ sensitivity to GLS-derived bitter taste. Interactions between taste modalities must also be considered; our results showed that all individual GSLs (except glucobrassicanapin) were negatively correlated with sweet taste. This was similarly reported by Francisco et al. [6], suggesting that bitter taste suppressed sweet taste in the perception of turnip.

Bitter taste was positively correlated with tannin flavour, and two individual GSLs were highly correlated with this attribute: 4-methoxyglucobrassicin and glucobrassicin. In our sensory profile data, batches B2, B6 and B7 were rated the highest in tannin flavour and bitter taste. The tannin flavour is likely to originate from phenolic compounds rather than from the GSLs. Such phenolic compounds (flavonoids, quinic acid derivatives, sinapic acids derivatives and tannins) have been found in turnip [39,40] and are also associated with bitter taste [36]. However, phenolic compounds were not measured in the current study, hence the relationship between bitter taste and phenolic compounds could not be determined.

It the present study, it was also observed that gluconapoleiferin, gluconapin, 4-hydroxyglucobrassicin, glucoerucin, glucobrassicin and gluconasturtiin and total GSL were highly correlated with earthy aroma, and gluconapoleiferin, glucobrassicin and 4-methoxyglucobrassicin were highly correlated with earthy flavour. In comparison, Helland et al. [33] observed that gluconapin, glucoerucin and glucobrassicanapin were positively correlated with earthy aroma. However, there are possible compounds other than GSLs that contribute to aroma and flavour of vegetables, such as the breakdown products of GSLs, which were not measured in this study.

## 5. Conclusions

The results obtained in this study showed that individual and total GSL varied between different batches of steamed-pureed turnip. The GSL compounds were correlated with aroma, taste and flavour characteristic of turnip. Almost all GSLs positively correlated with bitter taste; however, many GSLs correlated with other GSLs in concentration, which limits interpretation of which have the greatest influence on bitter taste. The strongest correlations with bitter taste were for 4-methoxyglucobrassicin and glucobrassicin; however, these two GSLs were highly correlated and the 4-methoxyglucobrassicin was at particularly low levels, so their individual contribution to bitter taste cannot be confirmed.

Overall, all batches of steamed-pureed turnip demonstrated both bitter and sweet taste, and these two taste characteristics were negatively correlated. It was evident that the bitter taste suppressed the sweet taste of the turnip as the batches containing the least GSL were the sweetest. The impact of this finding is in the conclusion that turnips bought commercially in the UK do provide a substantial amount of GSLs even after rigorous cooking and preparation, and as such cooked turnip could provide the well documented health benefits of GSLs if they were regularly consumed in the diet. However, the cooked product has a consistently bitter taste which may be a barrier to some consumers.

## Figures and Tables

**Table 1:** Purchase date and the origin of turnips for each batch.

Batch	Purchase Date	Origin of Turnip
**B1**	December 2015	The UK
**B2**	December 2015	The Netherlands
**B3**	February 2016	The UK (75%) and The Netherlands (25%) ^1^
**B4**	April 2016	The UK (24%) and The Netherlands (76%) ^1^
**B5**	April 2016	The UK
**B6**	June 2016	The Netherlands
**B7**	June 2016	The UK

^1^ For B3, 75% turnips came from the UK and 25% came from the Netherlands; for B4 24% turnips came from the UK and 76% came from the Netherlands.

**Table 2:** Definition of sensory characteristics associated with 7 batches of steamed-pureed turnip and references used during vocabulary development.

Sensory Characteristic	Definition
**Aroma**	
**Apple**	Aroma associated with apple
**Cooked swede**	Aroma associated with cooked swede
**Green vegetable**	Aroma associated with green vegetable (spinach)
**Sweetcorn**	Aroma associated with sweetcorn
**Savoury**	Aroma associated with savoury food
**Sweet**	Aroma associated with sweet food
**Earthy**	Aroma associated with earth or soil
**Starchy**	Aroma associated with starchy food (mashed potato)
**Tannin**	Aroma associated with tea
**Wet**	Aroma associated with musty
**Taste**	
**Salty**	Taste associated with sodium chloride
**Umami**	Taste associated with monosodium glutamate
**Sweet**	Taste associated with sucrose solution (0.5%, 1.0%, 2.0% and 2.6%)
**Bitter**	Taste associated with quinine sulphate solution (0.00005%, 0.0001%, 0.0002%, 0.0004% and 0.0006%)
**Flavour**	
**Earthy**	Flavour associated with earth or soil
**Tannin**	Flavour associated with tea
**Apple**	Flavour associated with apple
**Starchy**	Flavour associated with starchy food (mashed potato)

**Table 3:** Mean concentration of glucosinolates in seven batches of steamed-pureed turnip (B1 to B7). Results are expressed as μmolg-1 DW ± standard deviation. Different superscript letters indicate significant differences in mean concentration between batches. Abbreviation: ND, not detected.

Peak No.	Glucosinolate	Group	Side Chain	Mass Ion	Batch							*p* Value
					B1	B2	B3	B4	B5	B6	B7	
**1**	Progoitrin	Aliphatic	(2R)-2-hydroxy-3-butenyl	388	1.68 ± 0.03 ^ab^	1.94 ± 0.2 ^a^	1.73 ± 0.2 ^ab^	1.76 ± 0.04 ^ab^	1.34 ± 0.05 ^b^	1.37 ± 0.14 ^b^	1.69 ± 0.26 ^ab^	0.004
**2**	Glucoalyssin	Aliphatic	5-methylsulfinylpentyl	450	0.10 ± 0.09 ^ab^	0.14 ± 0.1 ^a^	ND ^b^	ND ^b^	ND ^b^	ND ^b^	ND ^b^	0.01
**3**	Gluconapin	Aliphatic	3-butenyl	372	1.15 ± 0.34 ^b^	2.03 ± 0.95 ^b^	1.22 ± 0.12 ^b^	0.80 ± 0.25 ^b^	0.43 ± 0.25 ^b^	9.49 ± 0.68 ^a^	11.21 ± 1.4 ^a^	<0.0001
**4**	4-hydroxy-glucobrassicin	Indole	4-hydroxy-3- indolylmethyl	463	0.32 ± 0.01 ^bc^	0.30 ± 0.02 ^c^	0.18 ± 0.03 ^d^	0.27 ± 0.01 ^c^	0.14 ± 0.01 ^d^	0.37 ± 0.03 ^ab^	0.39 ± 0.03 ^a^	<0.0001
**5**	Glucobrassicanapin	Aliphatic	4-pentenyl	386	3.77 ± 0.26 ^a^	5.06 ± 0.97 ^a^	4.76 ± 0.95 ^a^	3.70 ± 0.04 ^a^	1.92 ± 0.13 ^b^	1.29 ± 0.1 ^b^	1.33 ± 0.1 ^b^	<0.0001
**6**	Glucoerucin	Aliphatic	4-methylthiobutyl	420	0.48 ± 0.07 ^de^	0.84 ± 0.24 ^cde^	1.46 ± 0.14 ^c^	1.15 ± 0.55 ^cd^	ND ^e^	7.15 ± 0.32 ^a^	6.27 ± 0.39 ^b^	<0.0001
**7**	Glucobrassicin	Indole	3-indolylmethyl	447	0.87 ± 0.02 ^c^	1.08 ± 0.06 ^ab^	0.65 ± 0.06 ^d^	0.70 ± 0.08 ^cd^	0.90 ± 0.15 ^bc^	1.13 ± 0.04 ^a^	1.19 ± 0.07 ^a^	<0.0001
**8**	Glucoberteroin	Aliphatic	5-methylthiopentyl	434	1.37 ± 0.12 ^a^	1.56 ± 0.03 ^a^	0.95 ± 0.1 ^c^	1.08 ± 0.07 ^bc^	0.21 ± 0.06 ^d^	1.30 ± 0.09 ^ab^	1.38 ± 0.16 ^a^	<0.0001
**9**	Gluconasturtiin	Arylaliphatic	2-phenethyl	422	9.72 ± 0.27 ^bc^	10.94 ± 0.59 ^b^	8.96 ± 0.2 ^c^	9.20 ± 0.57 ^bc^	9.43 ± 0.1 ^bc^	19.81 ± 1.5 ^a^	19.32 ± 0.6 ^a^	<0.0001
**10**	4-methoxy-glucobrassicin	Indole	4-methoxy-3-indolylmethyl	477	0.05 ± 0.01 ^b^	0.07 ± <0.01 ^a^	0.03 ± <0.01 ^b^	0.04 ± <0.01 ^b^	0.05 ± <0.01 ^b^	0.07 ± 0.02 ^a^	0.05 ± < 0.01 ^ab^	<0.001
**11**	Glucona-poleiferin	Aliphatic	2-hydroxy-4- pentenyl	402	0.72 ± 0.01 ^e^	1.10 ± 0.02 ^cd^	1.00 ± 0.21 ^cd^	0.97 ± 0.04 ^d^	1.23 ± 0.01 ^bc^	1.38 ± 0.07 ^ab^	1.58 ± 0.06 ^a^	<0.0001
**12**	Neogluco-brassicin	Indole	N-methoxy-3- indolylmethyl	477	0.26 ± 0.03 ^b^	0.41 ± 0.03 ^a^	0.31 ± 0.06 ^b^	0.30 ± 0.01 ^b^	0.41 ± 0.03 ^a^	0.28 ± 0.02 ^b^	0.34 ± 0.02 ^ab^	<0.001
	Total glucosinolates				20.48 ± 0.67 ^bc^	25.46 ± 2.47 ^b^	21.25 ± 1.97 ^bc^	19.97 ± 1.47 ^bc^	16.07 ± 0.46 ^c^	43.64 ± 2.66 ^a^	44.74 ± 3.0 ^a^	<0.0001

**Table 4:** Mean scores for sensory characteristics for seven batches of steamed-pureed turnip. Different superscript letters indicate significant differences between batches.

Sensory Characteristic	Batch							Significance Between Samples (*p*-Value)
	B1	B2	B3	B4	B5	B6	B7	
**Aroma**								
**Apple**	2.8	4.3	8.0	4.0	9.1	3.8	2.8	0.34
**Cooked Swede**	15.7	17.6	13.4	20.8	15.4	21.9	22.3	0.21
**Green vegetable**	12.8	17.9	12.7	12.5	14.3	14.6	18.2	0.66
**Sweetcorn**	3.5	5.3	1.4	3.7	1.8	3.2	2.1	0.32
**Savoury**	18.0	24.0	19.8	21.6	22.8	24.9	26.3	0.06
**Sweet**	15.1	13.7	16.6	14.7	17.2	15.5	15.2	0.83
**Earthy**	11.0	12.5	9.7	11.2	9.5	16.6	20.1	0.06
**Starchy**	18.4	16.7	15.5	14.2	12.9	13.2	12.3	0.35
**Tannin**	2.1	1.8	2.0	2.4	2.4	1.3	2.9	0.75
**Wet**	12.2 ^ab^	14.7 ^a^	9.7 ^ab^	8.9 ^ab^	9.4 ^ab^	10.4 ^ab^	8.0 ^b^	0.04
**Taste**								
**Salty**	6.4	7.1	5.5	10.2	13.6	6.4	7.8	0.05
**Umami**	14.3	19.6	17.2	19.5	23.3	23.0	15.3	0.08
**Sweet**	33.1	30.3	31.2	35.3	30.5	34.5	26.3	0.24
**Bitter**	30.8 ^c^	53.2 ^a^	33.1 ^bc^	30.2 ^c^	34.5 ^bc^	40.3 ^bc^	43.3 ^ab^	<0.0001
**Flavour**								
**Earthy**	11.8	18.9	11.0	16.2	15.1	18.9	19.9	0.11
**Tannin**	9.8 ^b^	20.1 ^a^	8.3 ^b^	7.9 ^b^	9.4 ^b^	12.3 ^ab^	15.9 ^ab^	0.0003
**Apple**	4.3 ^abc^	3.3 ^bc^	8.7 ^abc^	12.0 ^ab^	12.6 ^a^	2.6 ^c^	1.9 ^c^	0.0008
**Starchy**	13.5	14.5	12.3	15.3	14.3	12.6	12.0	0.78
